# Unusual Inconsolable Crying: An Insight, Case Report, and Review of the Literature on the Pitt-Hopkins Gastrointestinal Phenotype

**DOI:** 10.7759/cureus.43781

**Published:** 2023-08-20

**Authors:** Francesco Comisi, Elena Esposito, Mariangela Marras, Consolata Soddu, Salvatore Savasta

**Affiliations:** 1 Pediatrics Department, Ospedale Microcitemico, Cagliari, ITA; 2 Radiology Department, Ospedale Microcitemico, Cagliari, ITA; 3 Genetics Department, Ospedale Microcitemico, Cagliari, ITA; 4 Pediatric and Rare Diseases Clinic, Ospedale Microcitemico, Cagliari, ITA

**Keywords:** pseudo-obstruction, abdominal pain, pathogenic variant, inconsolable crying, gastrointestinal obstruction, unusual cause of abdominal pain, pitt-hopkins syndrome

## Abstract

Pitt-Hopkins syndrome (PTHS) is a rare, neurodevelopmental genetic disorder caused by mutations in the TCF4 gene. This gene encodes a ubiquitous, class I, basic helix-loop-helix factor, which is implicated in various developmental and regulatory processes. Predominant clinical manifestations of PTHS include facial dysmorphisms, intellectual disability, absence of expressive language, epilepsy, as well as visual and musculoskeletal impairments. Gastrointestinal (GI) complications, such as chronic intestinal pseudo-obstruction, gastroparesis with delayed bowel transit, chronic constipation culminating in failure to thrive, and gastroesophageal reflux disease (GERD), are also prevalent in these patients. The early identification of pain etiology in PTHS patients poses a significant clinical challenge. This report presents two cases of PTHS patients suffering from gastrointestinal dysmotility, evaluated at our Pediatrics Clinic at the "Microcitemico" Hospital. A review of existing literature was conducted via the PubMed database to elucidate the current understanding of the GI phenotype in PTHS. Twenty articles were deemed most relevant and selected for this purpose. In both patients, severe constipation and abdominal distension resulted in persistent agitation and inconsolable crying. These distress symptoms were completely ameliorated following prompt pharmacological intervention.

## Introduction

Pitt-Hopkins syndrome (PTHS, MIM #610954) is a rare, genetic, neurological disorder first described by Australian physicians Pitt and Hopkins in 1978 [1]. Prevalence is estimated to range from 1:225.000 to 1:300.000 [2]. *TCF4* was identified as the causative gene in 2007, and the syndrome was subsequently related to deletion or pathogenic variants. It is located on chromosome 18q21.2 and encodes transcription factor 4 (*TCF4*) [3,4], a ubiquitous class I basic helix-loop-helix factor. *TCF4* plays an essential role in several processes [5], including the development and regulation of the CNS during intrauterine life [2,3,6] as well as of the immune system [7,8], which may explain the heterogeneity of the disease. PTHS was found to be usually caused by “de novo” heterozygous mutations, ranging from point mutations to partial and total gene deletions. However, germline mutations cannot be excluded, thus justifying the utility of prenatal diagnostic tests in families with a previously affected child. The clinical phenotype of PTHS and its management has recently been the object of the First International Consensus Statement, which provided diagnostic criteria and treatment recommendations [2]. The facial phenotype is characteristic of a cupid bow upper lip, bitemporal narrowing, thin lateral eyebrows, and flared nasal alae. CNS abnormalities have been reported, with microcephaly, hypo/agenesia of the corpus callosum, wide ventricles, and posterior fossa anomalies. Other features include ataxia, severe intellectual disability, axial hypotonia, stereotypes, lack of expressive language, autism, and epilepsy [2]. Concerning the correlation between epilepsy and PTHS, 21 PTHS patients have been the object of an observational study, 40% had generalized and focal epilepsy while 14% manifested developmental and epileptic encephalopathies [9]. Nearly half of patients with PTHS have spells of abnormal breathing, including hyperventilation, which is sometimes followed by apnea with rapid onset of cyanosis [10]. Visual and musculoskeletal impairments are also frequently reported. Gastrointestinal motility disorders are common in these patients and abdominal pain, constipation, feeding intolerance, and gastroesophageal reflux are symptoms that may occur and can negatively impact the quality of life. The main aim of this paper is to deeply analyze the gastrointestinal phenotype in PTHS as an unusual cause of persistent, inconsolable crying. We present two patients with PTHS who suffered from severe abdominal bloating, constipation, and persistent crying.

## Case presentation

Case 1

**Figure 1:**
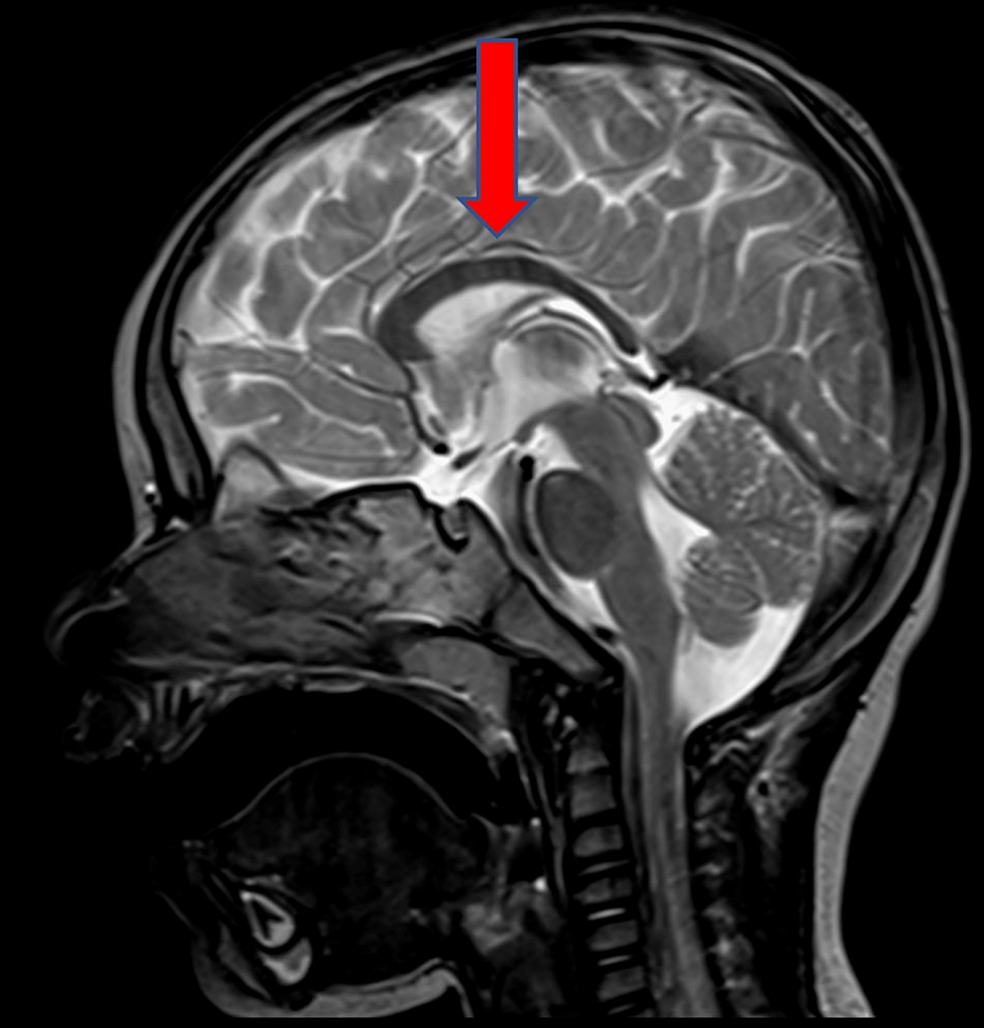
MRI sagittal T2W: thinning of the posterior body and splenium the of corpus callosum

The patient was a 12-year-old boy with PTHS caused by a “de novo” heterozygous p. Arg580Trp variant in the *TCF4* gene (c1738 C>T). He was born by normal pregnancy and delivery and non-consanguineous parents. Birth weight was 4040 g. At six months of age, infantile spasms were observed. EEG showed sporadic asymmetries with slow waves in the left temporal-central region and isolated sharp waves. Brain MRI revealed thinning of the corpus callosum (Figure 1).

**Figure 2:**
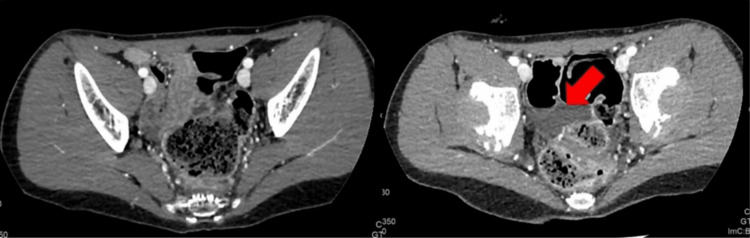
Post-contrast media abdomen CT: coprostasis and rectosigmoid distension with pelvic fluid effusion (arrow)

Clinical examination revealed a philtrum ridge, nasal bridge, a large mouth, anteverted nares, epicanthus, slightly curled helix, prominent metopic suture, hyperchromic “café-au-lait spots”, presacral dimple, pectus excavatum, mild dorsal kyphosis, and stereotypes. His developmental milestones were markedly delayed. He walked at four years old, his intellectual disability was severe, and expressive speech was absent; no further improvement was observed during the following years. At age 12 years, he was admitted to our clinic for acute pharyngitis and persistent crying in the previous seven days, without any evident trigger. Blood and urine analyses were normal. Abdominal ultrasound showed non-specific cecal appendix thickening. Abdominal distension and cramping abdominal pain were evident. He was persistently crying for more than 24 hours, and he decreased his oral intake. In order to exclude major emergencies, a CT scan was performed, revealing significant rectum and sigma distension with evidence of mechanical obstruction with fecal retention (Figure 2).

Oral therapy with macrogol together with lactulose was ineffective. During the following days, because of his extreme distress and agitation, repeated enemas were needed, leading to a significant improvement in abdominal distension.

Case 2

**Figure 3:**
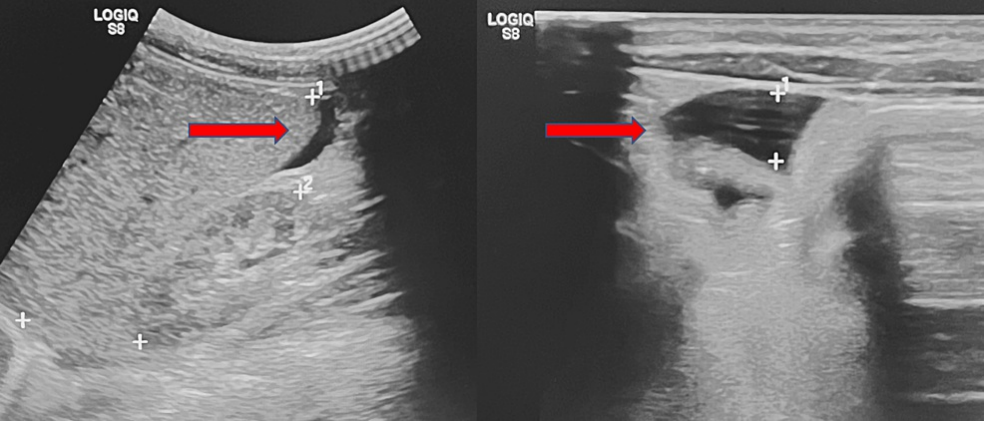
Abdominal US: intra-abdominal effusion along the hepatic margin and between the bowel loops

A three-year-old girl with PTHS, caused by a heterozygous mutation in the *TCF4* gene on chromosome 18q21, came under our care as she presented with severe abdominal pain, food refusal, constipation, and fever in the previous seven days. She was born by normal pregnancy and delivery, with non-consanguineous parents, and a birth weight of 3130 g. On clinical examination, she showed telecanthus, nasal bridge, short philtrum, small hands, anteriorized anus, severe psychomotor delay, absence of expressive language, and dysphagia. Persistent, inconsolable crying, abdominal distension, and mild fever were her main symptoms. Blood tests revealed moderate leukocytosis and abnormal electrolyte values. Urine cultures identified Escherichia (E.) coli pyelonephritis as the cause of fever and WBC elevation. Antibiotic therapy was immediately started, with the resolution of fever and leukocytosis. Despite the resolution of the urinary tract infection and the normalization of the blood values, abdominal bloating persisted, together with severe agitation, constipation, and food refusal. During the day, the crying was persistent due to abdominal distension. Transabdominal ultrasound revealed fluid between the intestinal loops (Figure 3).

**Figure 4:**
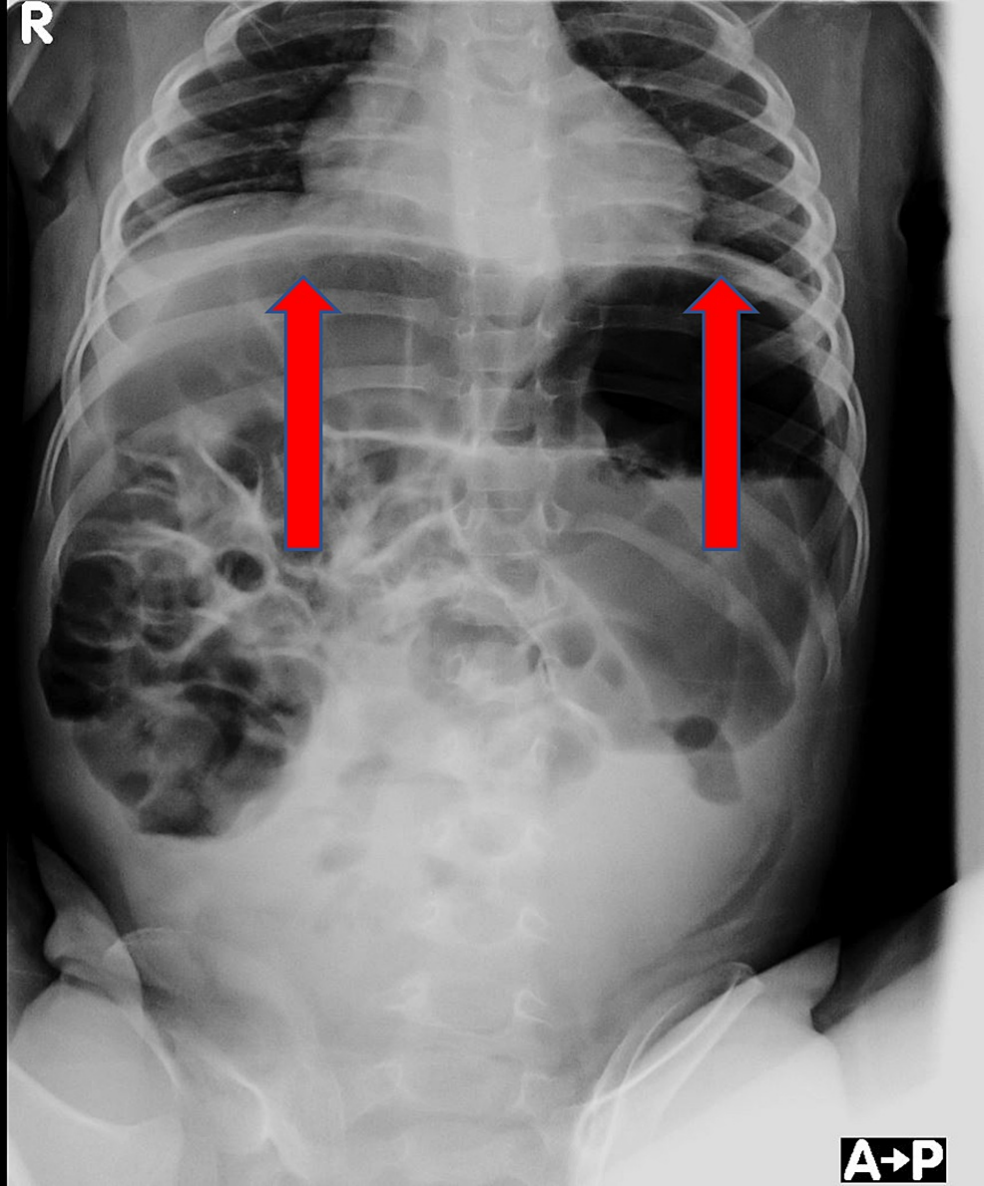
Abdomen X-ray: in the upper abdominal quadrants, massive dilatation of the large and small bowel causes the elevation of the diaphragm

Further abdominal X-ray showed severe colic distension, causing diaphragmatic elevation (Figure 4).

Continuative therapy with macrogol was administered, followed by a rectal probe and enemas, which solved constipation and distress. 

## Discussion

Gastrointestinal symptoms are very common in PTHS patients and often present as severe abdominal distension and chronic constipation. Some children may experience difficulty in breastfeeding due to axial hypotonia characterizing early childhood, which usually resolves in adulthood. Over the years, some will be able to feed independently while others will need to be fed. According to Zollino et al.’s First International Consensus Statement, common gastrointestinal disorders in PTHS include constipation (80%), gastroesophageal reflux disease (GERD) (38%), and burping [2]. Goodspeed and colleagues report similar data, with constipation being present since infancy in 70% of the considered cases [10]. As a result of autonomic abnormalities, affected subjects present with hyperventilation. We can assume that the increased intake of air can result in aerophagia and abdominal pain, up to severe complaints. This is the case of a patient who was reported by Zollino et al. and whose symptoms were solved after gastrostomy. Some children have also experienced pyloric stenosis and intestinal malrotation, the latter with a frequency of 19% of the cases analyzed by Goodspeed et al. [10]. Only a patient affected by Hirschsprung disease with symptoms of defective intestinal transit during the first days of life is reported [11]. Koppen and colleagues reported the case of two young adults with a history of constipation and abdominal distension, markedly worsening their quality of life, who died because of gastrointestinal complications likely related to PTHS [12]. To date, the cause of GI symptoms is not fully understood. A relevant contribution was given by Grubišić et al. in 2015, who demonstrated an altered GI transit in a mouse model. They showed that murine models had reduced upper GI and distal colon transit velocities, providing a fundamental contribution to the understanding of gastrointestinal symptoms [13]. In patients affected by PTHS, early detection of the causes of pain is extremely important.

Pitt-Hopkins syndrome is a rare and complex disease. Patients have poor or absent expressive language and are often non-verbal, so promptly recognizing the origin of their pain can be challenging. Based on our experience and literature review, we strongly recommend performing a targeted and tailored gastrointestinal follow-up. Proactive care may surely play a fundamental role. A daily diary of evacuation habits, drawn up by the patient’s caregiver, could help in the early interception of an intestinal obstruction as suggested by Zollino et al. Pediatricians should provide adequate training regarding the normal rate of evacuations, consistency of stools, and Bristol scale and be sure caregivers fully understand the importance of this type of monitoring, in order to reduce pain and hospitalization. As proposed by Watkins and colleagues [14], measures such as the Face, Legs, Activity, Cry and Consolability (FLACC) behavioral pain assessment scale and the Non-Communicating Children’s Pain Checklist (NCCPC) [15] may be used to explore the relationship between pain and behavior in PTHS. Considering the altered gut motility, permanent therapy with osmotic laxatives, stool softeners, and a diet rich in fibers may be taken into consideration. Recently, Aquino et al. reported the case of a 10-year-old child affected by severe constipation and treated with osteopathic manipulative sessions [16]. This type of approach showed good results in terms of evacuation frequency and reduction of enema administration but further studies with a larger cohort are needed to define its actual effectiveness. Transabdominal ultrasound should be part of the periodic follow-up and the first investigation to perform for acute and unexplainable pain in a PTHS patient. Constipation is indeed one of the most frequent symptoms of the syndromic phenotype, and recent evidence suggests a strong correlation of an enlarged ultrasound transrectal diameter with constipation [17-20]. Adherence to this approach, we think, may help healthcare professionals to timely detect and treat constipation-related severe distress and improve PTHS patients’ lives.

## Conclusions

In a child with Pitt-Hopkins syndrome (PTHS), persistent agitation and inconsolable crying could potentially signal pseudo-obstruction, necessitating immediate clinical attention. Therapeutic strategies, including enemas, osmotic laxatives, stool softeners, and a fiber-rich diet, may alleviate these symptoms. Incorporating regular transabdominal ultrasounds into routine clinical practice could facilitate early detection and treatment of distress. The primary objective here is to heighten the level of awareness among medical practitioners, enabling them to carry out exhaustive screening for gastrointestinal symptoms pertinent to PTHS patients. By leveraging such integrated healthcare tactics, the diverse and complex needs of PTHS patients can be efficiently addressed. Therefore, promoting professional cognizance and fostering comprehensive care models can notably ameliorate patient outcomes in PTHS.
